# Development of an aptamer-conjugated fluorescent nanoprobe for MMP2

**DOI:** 10.1186/1556-276X-9-104

**Published:** 2014-03-03

**Authors:** Myoung-Eun Han, Sungmin Baek, Hyun-Jung Kim, Jung Hwan Lee, Sung-Ho Ryu, Sae-Ock Oh

**Affiliations:** 1 Department of Anatomy, School of Medicine, Pusan National University, 49 Busandaehak-ro, Mulgeum-eup, Yangsan 626-870, Republic of Korea; 2 Medical Research Center for Ischemic Tissue Regeneration, Pusan National University, 49 Busandaehak-ro, Mulgeum-eup, Yangsan 626-870, Republic of Korea; 3 Medical POSTECH Aptamer Initiative Program, Division of Integrative Bioscience and Biotechnology, Pohang University of Science and Technology, Pohang 790-784, Republic of Korea

**Keywords:** MMP2, Aptamer, Nanoprobe, Atherosclerosis, Cancer

## Abstract

Matrix metalloproteinase 2 (MMP2) plays critical roles in various diseases, such as atherosclerosis and cancer, and has been suggested to contribute to the instability of atherosclerotic plaque. To visualize MMP2 in pathologic tissues, we developed an aptamer targeting MMP2 protein by performing eight rounds of modified DNA systematic evolution of ligands by exponential enrichment (SELEX). The aptamer showed high affinity for MMP2 (*K*_d_ = 5.59 nM), precipitated MMP2, and detected MMP2 protein in pathological tissues such as atherosclerotic plaque and gastric cancer tissues. Furthermore, a MMP2 aptamer-conjugated fluorescent nanoprobe successfully visualized atherosclerotic plaques in apolipoprotein E (ApoE) knockout mice. These results suggest that the devised MMP2 aptamer could be useful for the development of various diagnostic tools.

## Background

Matrix metalloproteinases (MMPs) are zinc- and calcium-dependent proteolytic enzymes [1,2]. MMPs can digest extracellular matrix proteins, such as collagen and fibronectin, and many other proteins, such as proteinases, growth factors, cytokines, chemokines, and cell receptors and thus regulate their activities. MMP was first identified in 1962 [3], and since then, other MMPs have been identified. Interestingly, whereas many MMPs are secreted by cells, others are anchored on cellular membranes. Members of this family play important roles in various cellular processes, such as migration, differentiation, and proliferation. Furthermore, they have been associated with pathophysiologies of various diseases, such as cancer, atherosclerosis, and arthritis.

During the progression of atherosclerosis, inflammatory cells such as monocytes and lymphocytes [4] play critical roles. Monocytes are recruited into atherosclerotic sites and differentiate into macrophages. After excessive lipid uptake, they become foamy cells. Notably, plaque macrophages secrete critical molecules such as MMPs and prothrombotic tissue factor. Then, MMPs destabilize atherosclerotic plaque by degrading extracellular matrix [5,6]. In addition to the roles in atherosclerosis, MMPs can aid the metastasis of cancer cells [2,7].

Information about the stability of atherosclerotic plaque is critical for the stratification and management of patients [8], and unfortunately, anatomical imaging modalities, such as CT or MRI, do not provide this type of information. Because MMPs are associated with the stability of atherosclerotic plaque, their visualization will be helpful in the stratification and management of patients.

The above mentioned actions of MMPs encouraged us to develop a molecular imaging modality that specifically targets MMP molecules. As compared with antibodies, aptamers have several beneficial characteristics, such as low immunogenicity, low molecular weight (8 to 15 kDa), high stability, better penetration, high affinity, and ease of production [9]. From these reasons, we decided to develop a MMP2-specific aptamer. By performing modified DNA systematic evolution of ligands by exponential enrichment (SELEX), we successfully developed a MMP2-specific aptamer which had high affinity and specificity and showed the possibility that it can be applied for molecular imaging.

## Methods

### *In vitro* selection of MMP2 DNA aptamers

To select MMP2-specific aptamers, a modified DNA SELEX procedure was used, as previously described [10]. Briefly, an ssDNA library template consisting of a 40-nucleotide random region (N40) flanked by two constant regions was prepared and immobilized on streptavidin-coated beads (Pierce, Rockland, MA, USA) via its 5′–OH-end biotin. A primer extension was then performed using the dATP, dCTP, dGTP, and benzyl-dUTP nucleotides. The modified DNA library was detached from the template under high pH conditions and then incubated with biotin-tagged target, partitioned using Dynabeads MyOne (Invitrogen, Carlsbad, CA, USA) and amplified by conventional PCR using a 5′–OH terminal biotinylated reverse primer. A primer extension was then performed, and an enriched pool was prepared for the next round. After eight rounds of SELEX, the enriched DNA pool was cloned and sequenced using standard procedures. After each round of SELEX, binding assays were performed to measure the dissociation constant (*K*_d_) value of the aptamer pool to ensure that its *K*_d_ value exhibited a decreasing trend.

### Binding assay

MMP2 aptamers were assayed for their ability to bind recombinant MMP2 (R&D Systems, Minneapolis, MN, USA). Aptamers were end-labeled with [*α*-32P]ATP and heated at 95°C for 3 min and then slowly ramped to 37°C at 0.1°C/s in buffer (40 mM HEPES (pH 7.5), 120 mM NaCl, 5 mM KCl, 5 mM MgCl2, 0.002% tween-20) for aptamer refolding. Aptamers were then incubated with purified MMP-2 at various concentrations for 30 min at 37°C. In order to capture MMP-2, the solution was incubated with Zorbax silica beads (Agilent, Santa Clara, CA, USA) for 1 min with shaking. The protein bead complex was then partitioned through nitrocellulose filter plates (Millipore, Billerica, MA, USA), which were then washed in buffer and exposed to photographic film. Amounts of radiolabeled aptamer that interacted with proteins were quantified using a Fuji FLA-5000 Image Analyzer (Tokyo, Japan). Dissociation constants were calculated by plotting bound MMP2 aptamer versus protein concentration using the following equation: *Y* = *B*_max_*X*/(*K*_d_ + *X*), where *B*_max_ is the extrapolated maximal amount of bound aptamer/protein complex.

### Precipitation of MMP2 protein by the aptamer and western blotting

A protein solution containing MMP2 protein (R&D Systems, Minneapolis, MN, USA) was precleared with streptavidin-coupled beads for 2 h. The solution was then precipitated with biotin-labeled MMP2 or control aptamer at 4°C overnight. Beads were washed four times with 1 ml of wash buffer (200 mM Tris at pH 8.0, 100 mM NaCl and 0.5% NP-40), once with ice-cold phosphate buffered saline (PBS), and boiled in 2× loading buffer. Finally, proteins were resolved by SDS-PAGE before being probed with MMP2 antibody (AB37150, Abcam, Cambridge, England, UK).

### Immunohistochemistry

Tissue samples were embedded in OCT (Sakura, Japan), and 4-μm thin sections prepared using a cryostat (CM3050S, Leica, Wetzlar, Germany) were placed on poly-lysine-coated microscope slides. The sections were then treated with 0.3% hydrogen peroxide for 30 min to quench endogenous peroxidase activity. Blocking was performed using 10% normal donkey serum (NDS) in 1× PBS. MMP2 aptamer or anti-MMP2 antibody (AB37150, Abcam, Cambridge, England, UK) binding was performed at a dilution of 1:200 in blocking buffer overnight at 4°C, and secondary antibody (horseradish peroxidase-conjugated anti-rabbit, 1:5,000) binding was performed for 2 h at RT. The signal was detected with HRP (Jackson ImmunoResearch Laboratories, West Grove, PA, USA) using the DAB substrate kit (Vector Laboratories, Burlingame, CA, USA). Sections were then counterstained with hematoxylin, dehydrated, and mounted. The primary antibody was omitted from negative control.

### Construction of an aptamer-conjugated nanoprobe

An aptamer-conjugated nanoprobe was produced as previously described [11]. MNP@SiO2(RITC)-(PEG)/COOH/pro-N/NH2 nanoprobes (MF nanoparticles, 2 mg/mL) were purchased from Biterials (Seoul, Korea). The carboxyl moieties (1.1 × 10^4^/nanoparticle) of MF nanoparticles (size, approximately 50 nm; hydrodynamic diameter, 58.1 nm) were covalently linked to a 5′-NH2-modified MMP2 aptamer using N-(3-dimehylaminopropyl)-N-ethylcarbodiimide (EDC) (Sigma, St. Louis, MO, USA). After 1 h of incubation, the aptamer-conjugated nanoprobe was washed twice with Tris buffer (pH 7.4) and briefly sonicated.

### Animal experiments and *ex vivo* imaging

To induce atherosclerosis in mice, apolipoprotein E (ApoE) knockout mice (Jackson Lab, Bar Harbor, ME, USA) were fed with a high cholesterol diet for 16 weeks from 8 weeks of age. All mice were housed under specific pathogen-free conditions in box cages at 23°C ± 2°C and 60% ± 10% humidity under a 12-hlight/12-h dark cycle with free access to food and water. Mice were sacrificed at week 16 of the experimental period. All animal procedures were performed in compliance with the Institute of Laboratory Animal Research Guide for the Care and Use of Laboratory Animals and approved by the Institutional Animal Care and Use Committee of Pusan National University. Atherosclerotic plaques were visualized by oil red O staining (Sigma). Aortas were removed 2 h after intravenously injecting MMP2 aptamer-conjugated fluorescent nanoprobe. Fluorescence from aortas was observed with Optix MX3/Optical Molecular Imaging System (ART, Montreal, Canada).

## Results and discussion

**Figure 1:**
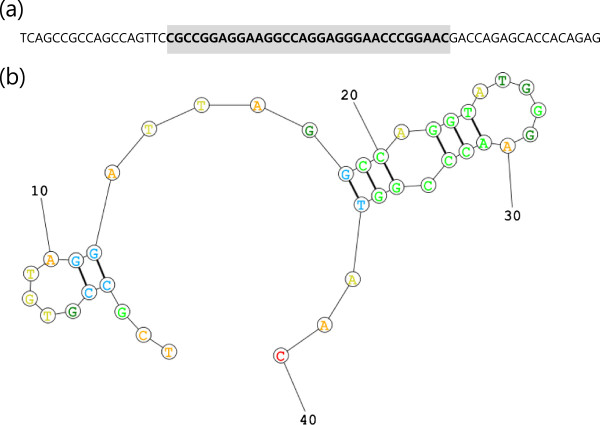
**Sequence and secondary structure of the MMP2 aptamer. (a)** Sequence of the 40-nucleotide random region (N40, shaded) and of the two constant regions flanking the random region. **(b)** The hairpin-like secondary structure of the aptamer is presented in the lower panel.

**Figure 2:**
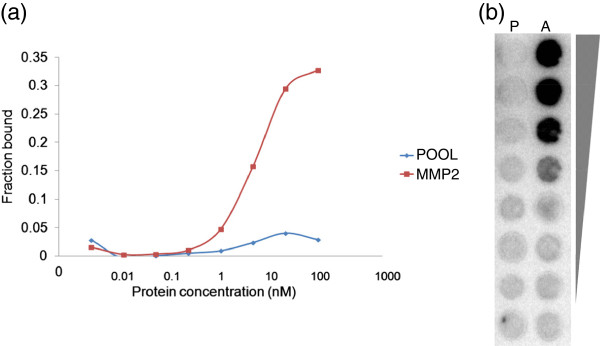
**Affinity of the MMP2 aptamer. (a) **^32^P-labeled aptamers and different MMP2 protein concentrations were used to examine the binding affinity of the MMP2 aptamer. **(b)** Images of radiolabeled aptamer that interacted with proteins in the binding assay.

To develop a specific aptamer for MMP2 protein, we performed a modified DNA SELEX technique as described in the ‘Methods’ section. To select a high-affinity aptamer, we used nucleotides chemically modified by benzylaminocarbonyl-dU (Benzyl-dU) at the 5′ positions, which mimic amino acid side chains. After eight rounds of SELEX, the enriched DNA pool was cloned and sequenced according to standard procedures. After each round of SELEX, binding assays were performed to measure the dissociation constant (*K*_d_) value of the aptamer pool using [*α*-32P] ATP. The sequence and secondary structure of the best aptamer selected in this study were presented in Figure 1. The mean *B*_max_ and *K*_d_ values of the aptamer were 35% ± 0.8% and 5.59 ± 0.52 nM, respectively (Figure 2).

**Figure 3:**
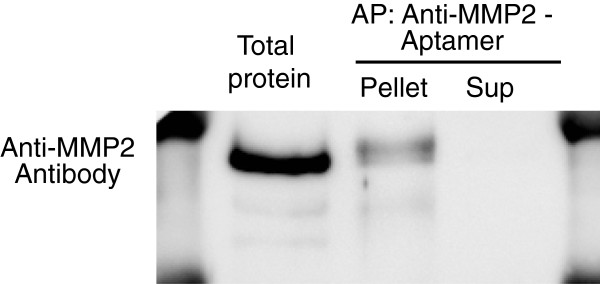
**Precipitation of MMP2 protein by MMP2 aptamer.** MMP2 protein in buffer containing 10% serum was incubated with the aptamer (0.2 μg/ml) overnight at 4°C. The protein was detected by immunoblotting with anti-MMP2 antibody.

To determine whether the MMP2 aptamer could be used to precipitate the target protein, we performed precipitation and then western blotting using anti-MMP2 antibody. To do this, we biotinylated the aptamer and used streptavidin beads for the precipitation. MMP2 in buffer containing 10% serum was incubated with the biotinylated MMP2 aptamer, and the protein-aptamer complex was then precipitated and detected by immunoblotting. The aptamer successfully precipitated MMP2 protein (Figure 3), whereas the biotinylated control aptamer did not (data not shown).

**Figure 4:**
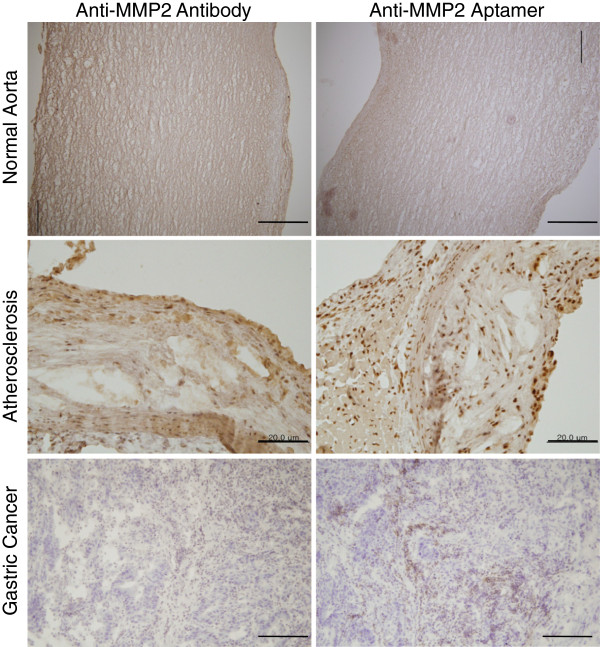
**Comparison of the tissue staining abilities of anti-MMP2 antibody and MMP2 aptamer.** Normal aorta, atherosclerotic plaques, and gastric cancer tissues were stained with anti-MMP2 antibody and MMP2 aptamer. Similar staining patterns were observed. Scale bar, 100 μm.

**Figure 5:**
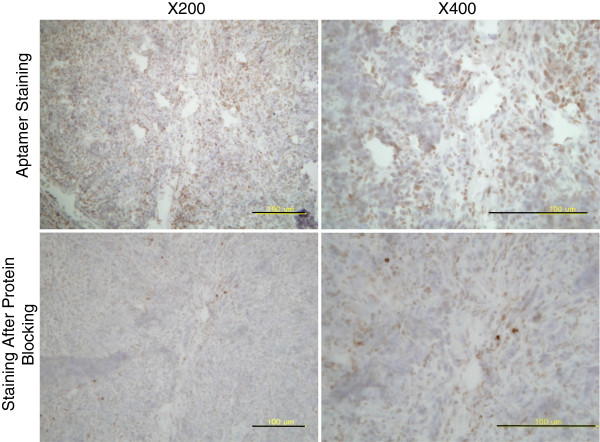
**Specificity of the aptamer by immunohistochemical staining.** After incubating the MMP2 aptamer with MMP2 protein in PBS at room temperature for 2 h, the immnohistochemical staining in gastric cancer tissues was significantly reduced. Scale bar, 100 μm.

Next, we examined whether the MMP2 aptamer could be applied for immunohistochemical purposes in pathological tissues, that is, atherosclerotic plaques and gastric cancer tissues. In both tissue types, the MMP2 aptamer successfully detected MMP2 (Figure 4), whereas the control aptamer did not (data not shown). To further confirm the specificity of the aptamer for immunohistochemistry, we performed peptide blocking. Immunohistochemistry was performed after incubating the aptamer for 2 h with the bare protein, and the intensities of positive signals were significantly reduced (Figure 5).

**Figure 6:**
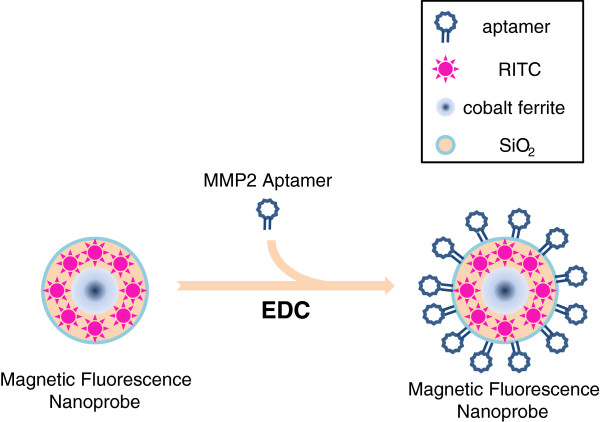
**Construction of the MMP2 aptamer-conjugated fluorescent nanoprobe.** The MMP2 aptamer was conjugated into magnetic fluorescent nanoprobe using EDC.

**Figure 7:**
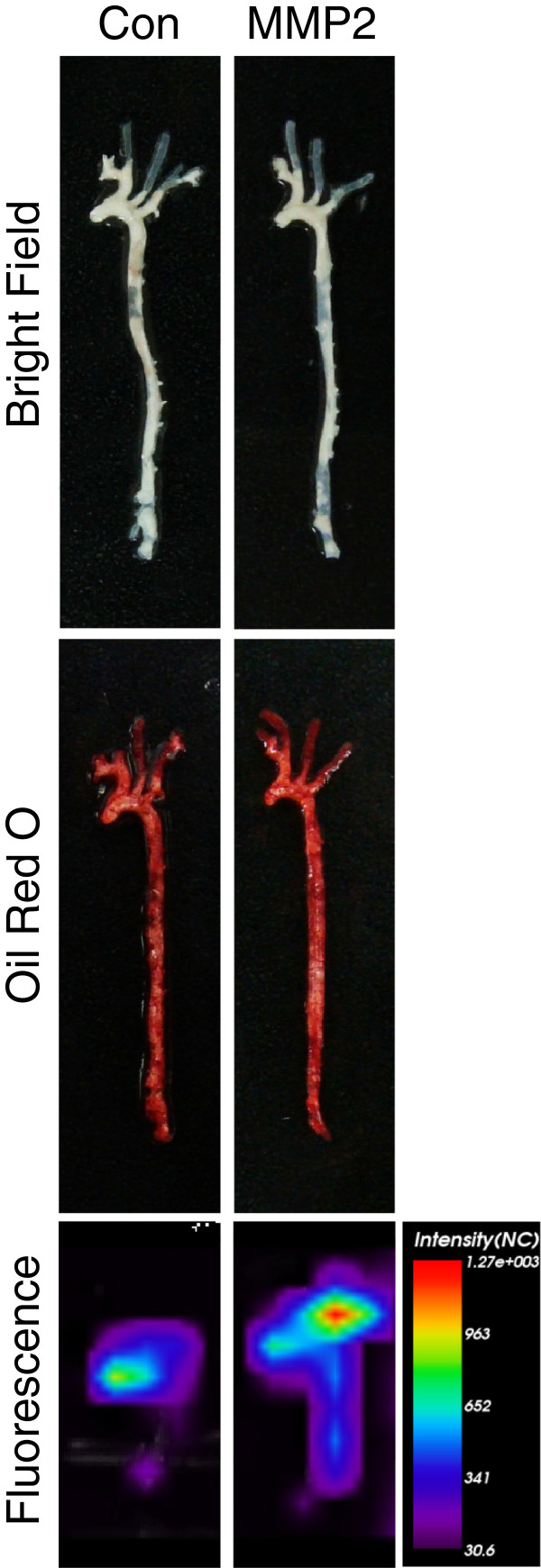
***Ex vivo *imaging of atherosclerotic plaques using the MMP2 aptamer-conjugated fluorescent nanoprobe.** Atherosclerotic plaques were induced by feeding ApoE knockout mice a high cholesterol diet for 4 months and were confirmed by oilred O staining (middle panels). *Ex vivo* imaging was performed 2 h after intravenously injecting mice with the MMP2 aptamer-conjugated fluorescent nanoprobe. The MMP2 aptamer (right panels) showed much stronger signals in atherosclerotic plaques than the control aptamer (left panels).

Finally, we used the aptamer for *ex vivo* imaging. To do this, the aptamer was conjugated to fluorescent nanoprobe using EDC (Figure 6). To induce atherosclerosis in mice, ApoE knockout mice were fed a high cholesterol diet for 4 months. After injecting the aptamer-conjugated fluorescent nanoprobe into a tail vein, fluorescent signals from atherosclerotic plaques were observed. The presence of atherosclerotic plaques was confirmed by oilred O staining. The MMP2 aptamer-conjugated nanoprobe produced significantly stronger signals in atherosclerotic plaques than the control aptamer-conjugated probe (Figure 7).

Many studies have tried to visualize MMP molecules. Small molecular MMP inhibitors attached to radioisotopes, such as^123^I, ^99m^TC, and ^18^ F have been used for the imaging of atherosclerotic lesions and myocardial infarctions [12-15]. Notably, a peptide substrate, which fluoresces when cleaved by MMPs, was used to visualize MMP activity [16-18]. However, considerable time is required for *in vivo* imaging using this peptide substrate. We considered that aptamers could overcome this problem because aptamers bind directly to target proteins. In addition, due to its small size and easy chemical modification, it can be easily applied to construct new nanoparticles as presented in this study ([9], Figure 6).

The specificity of the MMP2 aptamer produced during the present study was confirmed *in vitro* and *ex vivo*. Precipitation and immunohistochemistry studies demonstrated specific protein binding by MMP2 aptamer, and in particular, immunohistochemical staining of MMP2 aptamer was blocked by MMP2 protein. Furthermore, *ex vivo* imaging demonstrated that whereas MMP2 aptamer visualized atherosclerotic plaques, control aptamer did not. These results suggest that the devised MMP2 aptamer has clinical merit.

## Conclusions

We developed an aptamer targeting MMP2 protein using a modified DNA SELEX technique. The devised MMP2 aptamer precipitated and detected MMP2 protein in pathological tissues, that is, atherosclerotic plaques and gastric cancer tissues. Furthermore, the MMP2 aptamer-conjugated fluorescent nanoprobe allowed the visualization of atherosclerotic plaques in ApoE knockout mice. These results indicate that the developed MMP2 aptamer provides a suitable basis for the development of diagnostic tools.

## Competing interests

The authors declare that they have no competing interests.

## Authors’ contributions

ME carried out conjugation of the aptamer into the fluorescent nanoprobe and all animal experiments and drafted the manuscript. SM carried out immunohistochemistry. HJ carried out western blotting and immunohistochemistry. JH and SH carried out SELEX. SO conceived of the study, participated in its design and coordination, and helped to draft the manuscript. All authors read and approved the final manuscript.
